# Draft Genome Sequence of FK506-Producing *Streptomyces tsukubensis* Strain VKM Ac-2618D

**DOI:** 10.1128/MRA.00510-19

**Published:** 2019-06-13

**Authors:** Veronika Y. Poshekhontseva, Eugeny Y. Bragin, Victoria V. Fokina, Victoriya Y. Shtratnikova, Irina P. Starodumova, Sergey V. Tarlachkov, Marina V. Donova

**Affiliations:** a G. K. Skryabin Institute of Biochemistry and Physiology of Microorganisms, Pushchino Scientific Center for Biological Research of the Russian Academy of Sciences, Federal Research Center, Pushchino, Russia; b Pushchino State Natural Science Institute, Pushchino, Russia; c Department of Bioengineering and Bioinformatics, M. V. Lomonosov Moscow State University, Moscow, Russia; d Branch of Shemyakin and Ovchinnikov Institute of Bioorganic Chemistry, Russian Academy of Sciences, Pushchino, Russia; e Pharmins, Ltd., R&D, Pushchino, Russia; University of Rochester School of Medicine and Dentistry

## Abstract

The 23-membered macrolide tacrolimus (FK506) is an important immunosuppressant that is widely used in the prevention of graft rejection and in the treatment of inflammatory skin diseases and immune diseases. We report here the draft genome sequence of the FK506 producer Streptomyces tsukubensis VKM Ac-2618D.

## ANNOUNCEMENT

Tacrolimus (FK506) is widely used as an immunosuppressant after medulla, kidney, and heart transplantation (1). The FK506 yield from Streptomyces tsukubensis VKM Ac-2618D was higher (2) than that of *S. tsukubensis* NRRL 18488^T^, a wild-type progenitor of the industrially used high-FK506-producing strains (3).

*S. tsukubensis* VKM Ac-2618D is a Gram-positive soil-dwelling bacterial strain. It was previously identified as *S. tsukubaensis* based on 16S rRNA gene sequence analysis and chemotaxonomic, morphological, and physiological characteristics (2, 4).

Genomic DNA extraction from *S. tsukubensis* VKM Ac-2618D was carried out as described (5) with the following modifications. The cells from one colony were subcultured from agar into 50 ml of nutrient medium containing 20 g/liter starch, 20 g/liter glucose, and 18 g/liter peptone at pH 7.0 on a rotary shaker (220 rpm) at 25°C for 48 h. Then, 50 ml of the same medium was inoculated with 10% of the cell suspension obtained, and a second cultivation step was performed for 24 h. Bacterial biomass was harvested by centrifugation at 8,000 × *g* for 10 min and washed twice with sterile saline. Then, the cells were suspended in 3 ml Tris-EDTA (TE) buffer (25 mM Tris-HCl [pH 8], 10 mM EDTA) and 1 mg/ml lysozyme. SDS and proteinase K were added to final concentrations of 1% and 400 μg, respectively, and the suspension was incubated for 1 h at 54°C. RNase was added to a final concentration of 50 μg/ml, and the mixture was incubated for 30 min at 54°C. DNA was purified by sequential extractions of the liquid phase with phenol and the phenol-chloroform-isoamyl alcohol (25:24:1 [vol/vol/vol]) and chloroform-isoamyl alcohol (24:1 [vol/vol]) mixtures and precipitated with 0.7 volume of isopropanol at room temperature at 8,000 × *g* for 15 sec. Precipitated DNA was collected by centrifugation (4,000 × *g*, 4°C, 10 min), washed twice with 70% ethanol, air dried, and resuspended in TE buffer.

Fragmentation of the genomic DNA was done by sonication with a Covaris S220 instrument. The short-read library containing DNA fragments of 300- to 400-bp insert lengths was prepared with a NEBNext Ultra II DNA library prep kit (Illumina). The library was sequenced on an Illumina HiSeq 2500 platform with a HiSeq rapid PE cluster kit v2 and a HiSeq rapid SBS kit v2 (500 cycles).

Default parameters were used for all software unless otherwise specified. The read quality was checked with FastQC v0.11.8 (6). Raw Illumina sequencing data were adapter trimmed using BBDuk v38.35 (7) (with the parameters ktrim, r; k, 23; mink, 11; hdist, 1; tpe; minlen, 30; and ref, adapters), and then filtered to remove the known Illumina artifacts, and PhiX using BBDuk v38.35 (7) (with the parameters k, 31; ref, artifacts, phix; and cardinality). To remove possible contamination by human DNA, the reads were mapped to masked versions of a human reference genome (hg19) and discarded if the identity exceeded 95% using BBMap v38.35 (7) with the following parameters: minid, 0.95; maxindel, 3; bwr, 0.16; bw, 12; quickmatch; fast; and minhits, 2. The remaining reads were trimmed by quality scores using BBDuk v38.35 (7) with the following parameters: qtrim, r; trimq, 18; and minlen, 30.

Clean sequencing data were assembled using SPAdes v3.13.0 (8) with the parameters --careful and --cov-cutoff auto, and the resulting contigs were discarded if their length was <200 bp. Genome annotation was performed with the NCBI Prokaryotic Genomes Automatic Annotation Pipeline (9). The average nucleotide identities based on BLAST (ANIb) and digital DNA-DNA hybridization (dDDH) values were calculated using JSpecies v1.2.1 (10) and the Genome-to-Genome Distance Calculator (GGDC) v2.1 (11), respectively.

A total of 3.6 × 10^6^ paired-end reads with 251-bp lengths (1.8 Gb) were obtained from the sequencing; 91.9% of the bases had a quality score of over 30. As a result, 3.5 × 10^6^ clean paired-end reads with an average 226-bp length (1.6 Gb) were assembled into 79 scaffolds with 202-fold coverage. The scaffold *N*_50_ value is 971,906 bp, and the largest scaffold is 1,270,971 bp. The genome size is 7.93 Mb with an average G+C content of 71.9%. A total of 6,264 protein-coding genes (1,227 of which are hypothetical proteins), 66 tRNAs, 23 complete or partial rRNAs, and 3 noncoding RNAs (ncRNAs) were predicted.

The ANI and dDDH values calculated between the genome sequence of VKM Ac-2618D and the genome sequence of the type strain of *S. tsukubensis* NRRL 18488 (AJSZ00000000) were 99.97% and 99.9%, respectively, which are significantly higher than the threshold values (95 to 96% ANI and 70% dDDH) for species delineation (10–12). The results confirm that strain VKM Ac-2618D belongs to the species *S. tsukubensis*.

As expected, the tacrolimus biosynthesis cluster was found in the genome of the strain VKM Ac-2618D. According to the alignment, 8 nucleotide substitutions and 2 indels were observed between the gene clusters of VKM Ac-2618D (GenBank accession no. SGFG01000008; region of interest, nucleotides 83700 to 166586) and the type strain NRRL 18488 (GenBank accession no. AJSZ01000908; complementary strand of region of interest, nucleotides 93310 to 10426), while 100% identity was found for VKM Ac-2618D with the corresponding sequence of the *Streptomyces* sp. strain KCTC 11604BP (GenBank accession no. HM116537; region of interest, nucleotides 8712 to 91598).

The data obtained are important for the development of genome-based improvement of the *S. tsukubensis* strain to achieve higher tacrolimus production rates.

### Data availability.

The raw reads have been deposited in the NCBI SRA under the accession no. SRR8649924, and the whole-genome shotgun project has been deposited in DDBJ/ENA/GenBank under the accession no. SGFG00000000. The version described in this paper is the first version, SGFG01000000.
